# Predicting Prognosis in Internal Medicine: A Short and Long-Term Mortality Comparison Analysis

**DOI:** 10.7759/cureus.21734

**Published:** 2022-01-30

**Authors:** Renato Guerreiro, Célia Henriques, Sara Trevas, Cláudio Gouveia, Marta Roldão, Inês Egídio de Sousa, Catarina Faria, Gonçalo Pimenta, Inês Araújo, Candida Fonseca

**Affiliations:** 1 Internal Medicine Department, Hospital São Francisco Xavier, Lisbon, PRT; 2 Internal Medicine Department, Heart Failure Clinic, Hospital São Francisco Xavier, Lisbon, PRT; 3 Nephrology Department, Hospital de Santa Cruz, Lisbon, PRT

**Keywords:** geriatric population, transition care, predictors of mortality, early and long-term mortality, internal medicine ward

## Abstract

Introduction

The marked increase in life expectancy seen in Portugal in the last five decades led to a change in the profile of patients being most commonly admitted in internal medicine wards. In deciding the best care for these patients, prognostication models are needed in order to reduce readmissions, mortality, and adequate care. We aimed to study short and long-term mortality and predictors of all-cause mortality, independently of cause admission, of patients admitted in an internal medicine ward.

Methods

This two-part, single-center study enrolled patients from October 2013 to October 2014 with a follow-up of 60 months.

Results

A total of 681 patients were included; the mean age was 75.86 years with 60.4% females. The most frequent comorbidities were anemia, hypertension, and renal impairment. More than half of the population died in the follow-up period (51.5%). Deaths were significantly higher in the first six months after discharge (53% of all deaths) and then decreased abruptly to 11.6% in the second half-year after discharge. Based on the multivariate logistic regression model, with age over 80 years, anemia and neoplasm were independent predictors of short-term (p<0.001, p=0.001, p<0.001, respectively) and long-term (p<0.001 for the three conditions) mortality. Heart failure (p=0.018) and diabetes (p=0.025) were also predictors of long-term mortality.

Conclusion

High mortality, mainly in the first six months after discharge, elicits strategies targeting transition of care and close follow-up in the first months, which can be the key to improving outcomes. Identification of patients at higher risk may help design realistic models aiming to improve care for this frail population and decrease morbimortality.

## Introduction

Over the last decades, an important demographic transition in the Portuguese population has been observed, affecting all society sectors, with health being one of the most implicated [1]. The new public health politics and advances in medical care led to a significant increase in life expectancy at birth, which ranges from 67 years in 1970 to 81 years in 2019 (14 years in less than half a century) [1-2]. Consequently, the Portuguese population over 75 years old almost quadrupled in 50 years; in 1960, this population group was 238121 individuals (2.6% of the general population), and in 2011, it rose to 961 925 (9.1% of the population) [3]. In the same way, in the European Union, it is expected that the population over 80 years old will triple from 2011 to 2060 [4].

As expected, the characteristics of the population being admitted to the hospital also changed significantly. These patients are mainly of older age and present multiple comorbidities with several years of evolution [5-6]. Likewise, the practice of Internal Medicine, characterized by an integrated approach to patient care, was significantly affected by these trends in patient population’ demographics [5,7]. Temido H et al. had studied an Internal Medicine ward population in two different periods, 1992 to 1994 and 2012 to 2014 [5]. In 20 years, the median age of the admitted population increased by 18 years (from 61 to 79 years), and the percentage of patients with more than six secondary diagnoses at discharge increased from 17.5% to 64.8% [5].

With this increased complexity of patients being assisted in Internal Medicine wards, issues regarding prognostication and the ability to create prediction models in order to better individualize treatment approaches, thus aiming at reducing unnecessary readmissions and reducing mortality have emerged. We can find some evidence in the literature addressing this issue, specifically regarding predictors of short and long-term mortality after hospital discharge [8-11]. However, all of these studies are centered in a population with a specific disease, thus lacking the complexity and multimorbidity that typically characterizes the population admitted in internal medicine wards. Furthermore, the long-term mortality of patients after discharge is insufficiently characterized in the literature.

The aims of this study were to characterize the population admitted to an Internal Medicine ward of a university hospital in Portugal, over one year, and to study short and long-term mortality and predictors of all-cause mortality, regardless of admission diagnosis.

## Materials and methods

This was a two-part, single-center, observational study. In part I, all consecutive patients who were admitted to an Internal Medicine ward between October 2013 and October 2014 were prospectively included. After the first part of the study, patients were retrospectively followed for 60 months. We define short-term mortality as six-month mortality after discharge and long-term mortality as five-year follow-up mortality. In part II, all follow-up data were collected retrospectively. The entire study was purely observational in design with no interventions applied as part of the study protocol.

Per patient, data were collected on the first hospitalization (index event): demographics (age, gender, and diagnosis at the time of admission), clinical characteristics (main diagnosis at discharge and most common comorbidities, specifically heart failure (HF), atrial fibrillation, hypertension, diabetes mellitus, chronic obstructive pulmonary lung disease (COPD), stroke, anemia, renal impairment, and neoplasm) and laboratory blood tests that were performed at admission (serum hemoglobin, ferritin, vitamin B12, and folic acid, creatinine and estimated glomerular filtration (rate by the Chronic Kidney Disease Epidemiology Collaboration - CKD-EPI). Renal impairment was classified according to Kidney Disease: Improving Global Outcomes (KDIGO) nomenclature [12]. Anemia was defined according to World Health Organization criteria: hemoglobin < 12 g/dL for females and < 13 g/dL for males.

The study was approved by the Institutional Ethics Committee of Centro Hospitalar de Lisboa Ocidental, and participants provided their signed informed consent before study inclusion.

Statistical analyses were performed using SPSS version 25 (IBM Corp., IBM SPSS Statistics for Windows, Version 25.0. Armonk, New York: IBM Corp).

Subject demographics and baseline characteristics were summarized. Continuous variables were summarized using descriptive statistics, including the number of observations (n), mean, and standard deviation (SD). Categorical variables were summarized using the number and percent of subjects. Normality of the continuous data was assessed using visual inspection of histogram plots, P-P plots, and Q-Q plots. Linearity was assessed using scatter plots. Continuous variables were compared using the student's t-test or Mann-Whitney-U test, depending on normality. Proportional differences were tested using χ2 statistics or Fisher's exact test where appropriate.

For all survival analyses, follow-up was truncated at a maximum of five years (60 months). The cumulative probability of the outcome was illustrated using the Kaplan-Meier method with significance testing using log-rank statistics. Univariate and multivariable predictors of mortality were assessed in Cox's proportional hazards models. Proportional hazards assumptions were verified graphically.

The model contained the baseline variables previously shown to predict mortality based on bibliographic research. Variables were chosen according to significant differences between the subgroups at baseline. Candidate variables with P-values of <0.2 in univariate analysis were included in the multivariate model. For analysis of influencing variables on six-month, one-year, three-year, and five-year survival, single predictor Cox regression analyses were followed by multivariable Cox regression analysis.

A two-tailed P-value of <0.05 was considered significant in the final models.

## Results

**Figure 1:**
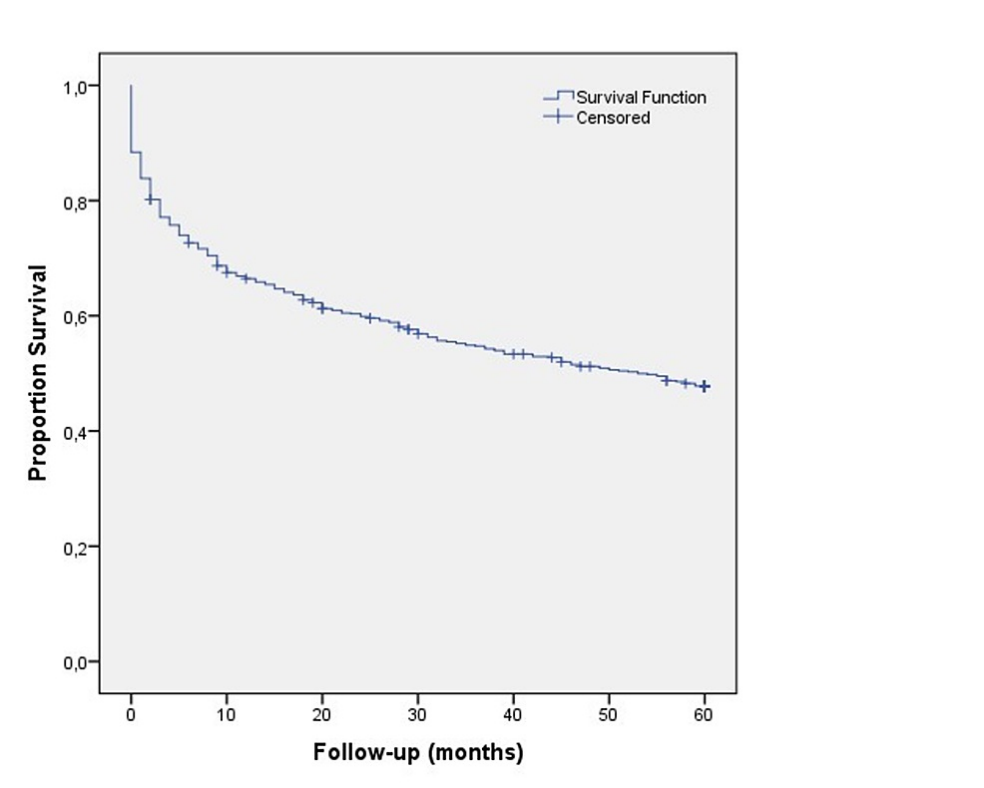
Cumulative survival curve

There were 681 patients enrolled, 30 were lost to follow-up. The demographic and clinical characteristics of the population at baseline are shown in Table 1. The mean age of the population was 75.86 (SD 14.17) years and 60.4% were females. The most frequent comorbidities were anemia (67.8%), renal impairment on admission (67.8%), hypertension (60.9%), iron deficiency (41.4%), and HF (33.6%). More than half of the population (351 individuals, 51.5%) died during the follow-up period (Figure 1). There was a significant difference between alive and deceased individuals, regarding age, during the follow-up period for some comorbidities: anemia, iron deficiency, HF, atrial fibrillation, stroke, COPD, neoplasm, and renal impairment at admission (Table 1).

**Table 1:** Baseline characteristics of the population admitted to the Internal Medicine ward: a comparison of the alive and dead population at the end of follow-up time COPD, Chronic Obstructive Pulmonary Disease; SD, Standard Deviation. ^a^at the end of follow-up time (60 months after admission); ^b^comparison between patients who were alive and dead at the end of follow-up; ^c^Kidney Disease: Improving Global Outcomes (KDIGO) nomenclature; ^d^statistical significance in expense in the moderate and severe groups

Variable	Total, n=681	Alive ^a^ n= 330 (48%)	Dead ^a^ n=351 (51,5%)	p-value ^b^
Age - mean (SD), years	75.86 (14.17)	69.95 (15.8)	81.41 (9.6)	< .001
Female sex, n (%)	411 (60.4)	199 (60.3)	212 (60.4)	.98
Anemia, n (%)	462 (67.8)	183 (55.5)	279 (79.5)	< .001
Iron deficiency, n (%)	282 (41.4)	115 (34.8)	167 (47.6)	< .001
Folate deficiency, n (%)	38 (5.6)	9 (3.2)	29 (7.2)	.065
Vitamin B12 deficiency, n (%)	39 (5.7)	17 (6.0)	22 (6.5)	.753
Renal impairment on admission^c^, n (%)	462 (67.8)	190 (57.6)	272 (77.8)	< .001^d^
Mild, n (%)	186 (27.3)	101 (30.6)	85 (24.2)	----
Moderate, n (%)	207 (30.4)	71 (21.5)	136 (38.7)	----
Severe, n (%)	69 (10.1)	18 (5.5)	51 (14.5)	----
Diabetes, n (%)	185 (27.2)	76 (23)	109 (31.1)	----
Heart failure, n (%)	229 (33.6)	78 (23.6)	151 (43)	< .001
Coronary ischemic disease, n (%)	67 (9.8)	27 (8.2)	40 (11.4)	.159
Atrial fibrillation, n (%)	156 (22.9)	61 (18.5)	95 (27.1)	.008
Hypertension, n (%)	415 (60.9)	189 (57.3)	226 (64.8)	.057
Stroke, n (%)	103 (15.1)	39 (11.8)	64 (18.2)	.020
Pulmonary embolism, n (%)	8 (1.2)	5 (1.8)	3 (0.8)	.424
COPD, n (%)	73 (10.7)	24 (7.3)	49 (14)	.005
Neoplasm, n (%)	111 (16.3)	22 (6.7)	89 (25.4)	< .001
Benign thyroid disease, n (%)	63 (9.2)	27 (9.6)	36 (9.1)	.592

Mortality was significantly higher in the first six months after discharge (182 people, 53% of all deaths), decreasing abruptly to 40 individuals (11.6% of all deaths) in the second half-year after discharge, and then the mortality rate reduced progressively from 5.9% individuals during the second year to 2.2% in the fifth year post-discharge (Figure 1).

On unadjusted analysis, short-term mortality (six-month mortality) and long-term mortality (five-year mortality) were compared and differed significantly for the following hospital admission co-morbidities: anemia (χ2 9.313, p=0.002), neoplasm (χ2 13.875, p<0.001), and renal impairment at admission (χ2 7.311, p=0.026). A history of neoplasm was the most statistically relevant variable associated with early mortality. There was a statistically significant difference in mortality between the two groups independently of admission cause (p<0.001).

At the six-month follow-up, in univariate analysis, there was a statistically significant association between death and female gender (p=0.036), age and death, but only for age > 80 years (p<0.001). Comorbidities associated with mortality were neoplasm (p<0.001), anemia (p<0.001), iron deficiency (p=0.017), and renal impairment at hospital admission (p=0.038). A negative correlation was detected between death and low hemoglobin values at admission (p<0.001) and a low estimated glomerular filtration rate (p<0.001).

Based on the Cox regression model, three variables were independent predictors of short-term mortality (Table 2): age over 80 years (HR=1.044, 95% CI 1.028-1.060, p<0.001), neoplasm (HR=3.056, 95% CI 2.196-4.252, p<0.001), and anemia (HR=1.951, 95% CI 1.307-2.910, p=0.001).

**Table 2:** Short and long-term independent predictors of mortality across the follow-up period HZ, Hazard Ratio; CI, Confidence Interval; COPD, Chronic Obstructive Pulmonary Disease; HF, Heart Failure

Time	Predictor
6 months	Neoplasm (HR 3.056; 95% CI 2.196-4.252; p < 0.001)
	Anemia (HR 1.951; 95% CI 1.307-2.910; p = 0.001)
	Age > 80 years (HR 1.044; 95% CI 1.028-1.060; p < 0.001)
1 year	Neoplasm (HR 3.108; 95% CI 2.304-4.191; p < 0.001)
	Anemia (HR 2.103; 95% CI 1.467-3.015; p < 0.001)
	COPD (HR 1.505; 95% CI 1.023-2.212; p = 0.038)
	Age > 80 years (HR 1.045; 95% CI 1.030 – 1.060; p < 0.001)
3 year	Neoplasm (HR 3.285; 95% CI 2.498-4.320; p < 0.001)
	Anemia (HR 1.932; 95% CI 1.425-2.621; p < 0.001)
	Diabetes (HR 1.329; 95% CI 1.020-1.733; p = 0.035)
	HF (HR 1.288; 95% CI 1.007-1.647; p = 0.044)
	Age > 80 years (HR 1.054; 95% CI 1.040-1.068; p < 0.001)
5 year	Neoplasm (HR 3.152; 95% CI 2.415-4.113; p < 0.001)
	Anemia (HR 1.730; 95% CI 1.321-2.264; p < 0.001)
	Diabetes (HR 1.325; 95% CI 1.035-1.696; p = 0.025)
	HF (HR 1.319; 95% CI 1.049-1.659; p = 0.018)
	Age > 80 years (HR 1.056; 95% CI 1.043-1.068; p < 0.001)

At the five-year follow-up, in univariate analysis, there was a statistically significant association between death and age but only for age > 80 years (p<0.001). Comorbidities associated with mortality were anemia (p<0.001), iron deficiency (p<0.001), neoplasm (p<0.001), anemia (p<0.001), iron deficiency (p=0.017), HF (p<0.001), neoplasm (p<0.001), renal impairment at hospital admission (p=0.004), stroke (p=0.020), and atrial fibrillation (p=0.008).

**Figure 2:**
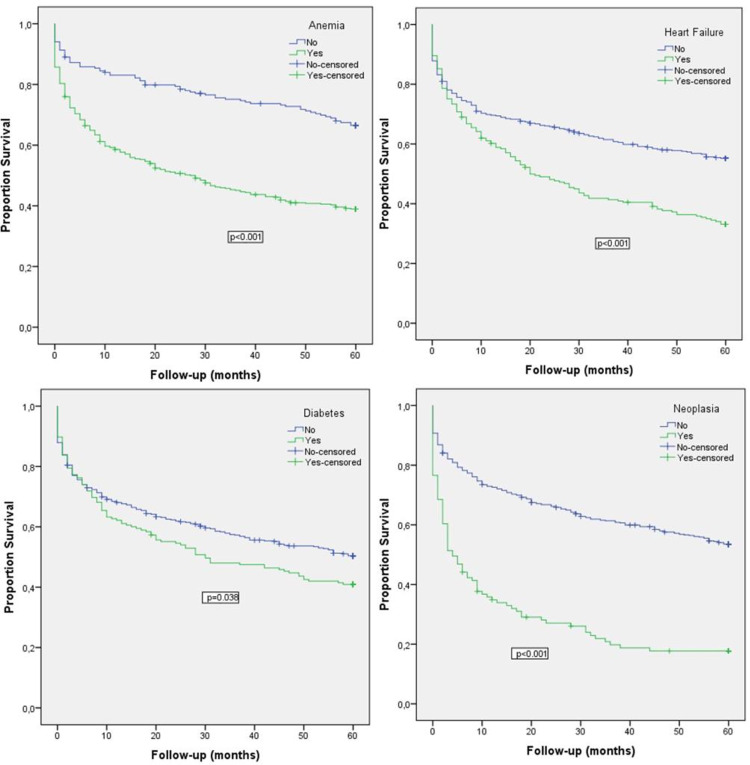
Cumulative survival curves by five-year predictors of mortality

Five variables were independently associated with long-term mortality (Table 2, Figure 2), namely, age over 80 years (HR=1.056, 95% CI 1.043-1.068, p<0.001), neoplasm (HR=3.152, 95% CI 2.415-4.113, p<0.001), anemia (HR=1.730, 95% CI 1.321-2.264, p<0.001), diabetes (HR=1.325, 95% CI 1.035-1.696, p=0.025), and HF (HR=1.319, 95% CI 1.049-1.659, p=0.018).

## Discussion

Prior studies showed important trends towards increased age and number of comorbidities in inpatients admitted to Internal Medicine wards. Based on this reality, clinical sense tells us that it is a population with very high mortality. However, there have been few studies conducted to characterize this population. We analyzed long-term mortality in a population admitted to an Internal Medicine ward. Short and long-term mortality predictors were investigated.

We confirm the previously published data on the older age of the current Internal Medicine wards' population (mean age 75.86 years in our study versus 75.6 years in the Temido H et al. study [5] and 74.2 years in the Marinho R et al. study [13]). The high number of comorbidities is also in agreement with Temido H et al.; 64.8% of their population had more than six versus 44.3% with five or more comorbidities in our population [5]. As shown by Verma A et al., hypertension, HF, diabetes, and atrial fibrillation are frequent comorbid conditions in the Internal Medicine population [14]. The same was reported previously for anemia by our group (67.8%) [15] and COPD by Tschopp J (9.1%) [16]. Neoplasm had also an important role in our study population (16.3%), reinforcing preceding data on the impact of neoplastic disease in this population. Pulido I et al. showed that 22.1% of patients deceased in an Internal Medicine ward had cancer as the primary admission diagnosis [17].

As reported in some chronic diseases, namely, HF, we confirmed very high mortality after discharge. Age, anemia, and neoplasm were predictors of death across short and long-term follow-up (Table 2). COPD was a predictor of one year after discharge mortality, diabetes, and HF for three and five years (Table 2). Despite scarce data on mortality after discharge from the Internal Medicine ward, there are available studies on mortality of specific chronic diseases.

Kaeberlein M in his article on “longevity and aging” refers to aging as the greatest risk factor for the main causes of death in developed countries, even more when associated with some other pathologies (heart disease, cancer, diabetes, and Alzheimer disease) [18]. In the same way, Ramírez S et al. identified the organic deterioration associated with aging as a determinant factor for death [4]. According to those authors' considerations, we also identified age as a short and long-term predictor of death in our elderly population.

Anemia is a major world health problem and the prevalence increases with age. The prevalence of anemia in older Portuguese adults was estimated at 21% for individuals over 65 years (17.3% between 65 and 79 years, 31.4% over 80 years) [19]. Anemia has been independently associated with short and long-term morbidity and mortality in hospitalized patients, especially in the elderly with concomitant heart or kidney disease, hypertension, or diabetes [4,20]. Our results identifying anemia as a predictor of death from six months to five years after discharge reinforce the previous literature data on the impact of anemia on mortality.

The neoplasm was another short and long-term independent predictor of mortality in our population. Suárez-Dono J et al. also identified neoplasm as a predictor of one and two-year mortality in patients' polypathology after hospital admission [21].

Argano C et al. reported a prevalence of 13.5% one-year mortality after hospitalization on an Internal Medicine ward of patients with COPD versus 14.1% for patients without COPD [22]. In our patients with COPD, mortality was 45.2% versus 32.1% in patients without COPD one year after discharge. Contrary to Argano C et al., COPD was a predictor of one-year mortality after discharge (Table 2).

HF is also a major health problem, with high rates of mortality and hospitalization on Internal Medicine wards [23]. Estimated mortality is around 20% one year after diagnosis and 50-60% five years after diagnosis [24]. HF was highly prevalent (33.6%) in our population and a predictor of mortality at three and five years after hospital admission. As previously reported by other authors, HF wasn’t a predictor of mortality at six months after discharge [25-26].

In our study, diabetes was another predictor of long-term mortality at three and five years after discharge. In a recent systematic review, Mukherjee et al. concluded that despite mortality being a central outcome for patients discharged with diabetes, few studies considered this outcome, and the conclusions are limited [27].

Regarding short-term mortality, our study highlights the increased risk in the first six months after discharge (26.7% mortality), comparable to the 29% reported by Sousa S et al. in 2002 on Portuguese population discharge from an Internal Medicine ward at a tertiary hospital [28]. These similar results suggest that in 11 years, from 2002 to 2013, we didn’t change clinical practices with an impact on six months mortality after discharge.

Persistent high six months mortality detected in our and other studies strongly suggests a central role for transition care and early post-discharge follow-up in this population, especially when neoplasm or cardiovascular disease are on board. The importance of transition care issue is growing, especially in the setting of chronic cardiovascular disease, COPD, and elderly populations, three factors related to vulnerability, readmissions, and mortality [24,29-30]. European guidelines on HF recommend a follow-up visit in one to two weeks after discharge to reduce early hospital readmissions and mortality [24]. The Health Quality Ontario group studied the effect of reevaluation of patients with HF and COPD, seven and 30 days after discharge in a systematic review and concluded that early follow-up reduced the risk of readmission and mortality [29]. Pedersen L et al. evaluated the impact of early follow-up on the geriatric population after admission to the emergency department, showing that this intervention reduced 30-day hospitalization but not mortality [30].

Our study results must be interpreted with caution since the study has some important limitations. Despite the prospective design of the first part of this study, the follow-up data regarding mortality were collected retrospectively. Admission causes were categorized into large diagnosis groups, with a big range of specific pathologies. Furthermore, we could not retrieve, with sufficient accuracy, the causes of mortality for the whole population due to unavailable data.

## Conclusions

The epidemiology of Internal Medicine wards has dramatically changed in the last decades. An older and more frail population with persistent high short and long-term mortality requires adaptation of health systems and practices.

High post-discharge mortality, mainly in the first six months, must foster the development of strategies targeting the transition of care and close follow-up in the first months after discharge, which can be the key to improving outcomes. Standardized care pathways post-discharge, specially built for patients at higher risk of early post-discharge mortality, are warranted. Action must be taken by physicians, institutions, and stakeholders in order to improve effectiveness, optimize care, and reduce costs.
